# Role of Archaeal HerA Protein in the Biology of the Bacterium *Thermus thermophilus*

**DOI:** 10.3390/genes8050130

**Published:** 2017-04-27

**Authors:** Alba Blesa, Nieves G. Quintans, Ignacio Baquedano, Carlos P. Mata, José R. Castón, José Berenguer

**Affiliations:** 1 Centro de Biología Molecular Severo Ochoa, Universidad Autónoma de Madrid-Consejo Superior de Investigaciones Científicas, Calle Nicolás Cabrera 1, Madrid 28049, Spain; ablesa@cbm.csic.es (A.B.); ngquintans@cbm.csic.es (N.G.Q.); ibaquedano@cbm.csic.es (I.B.); 2 Department of Structure of Macromolecules, Centro Nacional de Biotecnología (CNB), Consejo Superior de Investigaciones Científicas (CSIC), Cantoblanco, Madrid 28049, Spain; cperez@cnb.csic.es (C.P.M.); jrcaston@cnb.csic.es (J.R.C.)

**Keywords:** HerA, *Thermus*, DNA repair, transjugation, transformation, lateral gene transfer, hexameric ATPase

## Abstract

Intense gene flux between prokaryotes result in high percentage of archaeal genes in the genome of the thermophilic bacteria *Thermus* spp. Among these archaeal genes a homolog to the *Sulfolobus* spp. HerA protein appears in all of the *Thermus* spp. strains so far sequenced (HepA). The role of HepA in *Thermus thermophilus* HB27 has been analyzed using deletion mutants, and its structure resolved at low resolution by electron microscopy. Recombinant HepA shows DNA-dependent ATPase activity and its structure revealed a double ring, conically-shaped hexamer with an upper diameter of 150 Å and a bottom module of 95 Å. A central pore was detected in the structure that ranges from 13 Å at one extreme, to 30 Å at the other. Mutants lacking HepA show defective natural competence and DNA donation capability in a conjugation-like process termed “transjugation”, and also high sensitivity to UV and dramatic sensitivity to high temperatures. These data support that acquisition of an ancestral archaeal HerA has been fundamental for the adaptation of *Thermus* spp. to high temperatures.

## 1. Introduction

Phylogenetic analysis based on the use of 16sRNA and in concatenated analysis of up to 30 ribosomal protein genes, support that the genus *Thermus* belongs to one of the most ancient groups of bacteria, which also includes the radio-resistant mesophilic genera *Deinococcus* [1,2]. Further whole genome comparisons strongly support the close relationship between both genera, with a common ancestor likely of moderate thermophilic character that already contained genes transferred from thermophilic archaea and bacteria [3]. Further adaptation of the common ancestor of the clade to different extreme conditions required gene gain in the case of *Deinococcus*, which allowed its extreme resistance to radiation, and a balance between gene losses and gains in the case of adaptation to extreme temperatures for *Thermus* species (spp.). In the latter case, several genes have been acquired from thermophilic archaea, including many of them likely involved in DNA maintenance, DNA repair, and CRISPR-Cas defense systems [3]. Many of these archaeal genes are located in a megaplasmid (pTT27) present in most *Thermus* spp. which constitutes a very plastic element where most of the differences between the *Thermus* species concentrate [4].

A homolog of the HerA helicase is found among the genes of archaeal origin likely present in the common ancestor of the *Thermus-Deinococcus* clade [5]. In thermophilic archaea, the helicase HerA is an essential enzyme encoded in a UV-induced operon that includes a NurA nuclease and DNA repair protein homologs to the eukaryotic Mre11 and Rad50 proteins. These eukaryotic Mre11 and Rad50 counterparts recognize and bind to double-stranded breaks (DSB) before recruiting the nuclease and helicase components required to generate a 3’ single-stranded DNA overhang, which is further recognized by the Rad51 recombinase to initiate strand invasion in homologous recombination (HR). Thus, it is assumed that a similar role is played in archaea by HerA, NurA, and Mre11 and Rad50 homologs, which are co-translated from a polycistronic mRNA. In this context, a model for the concerted action of HerA and NurA has been recently proposed based on the crystal structure of the *Sulfolobus solfataricus* proteins in which the HerA hexamer pushes double-stranded DNA through its central channel towards a NurA dimer, where DNA degradation takes place [6].

The role of the archaeal HerA and NurA proteins has also been studied in *Deinococcus radiodurans* [7]. In this bacterium, the absence of *nurA*, *herA*, or both results in defective cell proliferation and a 10-fold reduced intermolecular recombination, but without apparent affection to the high radio-resistance shown by this organism. Two hybrid assays supported, for *D. radiodurans*, the existence of interaction between both proteins through the N-terminal HAS domain of HerA, with such interaction stimulating each other activity (ATPase for HerA and nuclease for NurA).

In all of the *Thermus* spp. so far sequenced, an archaeal HerA homologue is encoded in the chromosome. We will call this protein helicase protein A (HepA) hereafter to avoid confusion with the extensively studied dead-box RNA helicase Hera [8]. Here, we have investigated HepA both at the level of overall protein structure, through electron microscopy and image reconstruction, and also in the biology of *T. thermophilus.* Our data show that HepA plays a relevant role in adaptation to high temperatures as its absence severely affects UV resistance and impairs growth at high temperatures. Affection to DNA mobilization processes is also reported.

## 2. Materials and Methods

### 2.1. Strains and Growth Conditions

Bacterial strains and derived mutants are described in Table 1. *T. thermophilus* was routinely grown at 60 °C in Erlenmeyer flasks filled up to 1/5 of their capacity with *Thermus* broth (TB) under rotational shaking (150 rpm) [9]. *Escherichia coli* strains DH5α and BL21 (DE3) were used for cloning purposes and recombinant protein expression, respectively. Both strains and derivatives carrying plasmids were grown at 37 °C in liquid or solid LB media, with kanamycin (Km, 30 mg·L^−1^), ampicillin (Am, 100 mg·L^−1^), or hygromycin B (Hyg, 100 mg·L^−1^), as required.

### 2.2. Isolation of Mutants

Mutants were isolated by replacement of the targeted gene by a gene cassette encoding thermostable-resistant to Km through transformation of the parental strain with the appropriate linear DNA construction featuring upstream and downstream recombination arms [15]. PCR amplification with the oligonucleotides, described in Table 2, was used to obtain the recombination arms. The constructed plasmids are described in Table 3.

### 2.3. HepA Inmunodetection

The presence of the HepA protein was detected by Western blot on cell fractions of *T. thermophilus* cultures by using a rabbit antiserum raised against the purified protein. For this, an exponential culture of *T. thermophilus* HB8 was harvested, washed in phosphate buffer (50 mM sodium phosphate, pH 7), concentrated to 1/10 of the culture volume in the same buffer, and disrupted by sonication (4 °C, three pulses of 1 min with 0.5 s cycles, 0.9 intensity, Labsonic, Braun, Goettingen, Germany). Unbroken cells were discarded by centrifugation (5000× *g*, 5 min), and the soluble fraction was obtained after two consecutive centrifugation steps (15,000× *g*, 20 min). The particulate fraction, including membranes and cell wall polymers, was re-suspended in the same volume and buffer, and the SDS-soluble protein fraction was subsequently analyzed by PAGE and Western blot with anti-HepA antiserum. Goat anti-rabbit antibodies bound to horseradish peroxidase and chemiluminescence were used for detection (ECL, Amersham International, Buckinghamshire, UK).

### 2.4. Protein Purification and ATPase Assays

The gene *hepA* was cloned into plasmid pET28b, which adds a 6xHistidine tag fused to the N-terminus to generate pAB135. Expression in *E. coli* BL21 (DE3) cells was induced with 1 mM IPTG and growth was maintained for 4 h at 30 °C, after which cell pellets were harvested by centrifugation (23,700× *g*, 20 min at 4 °C) and broken by a French press (GEA Niro Soavi Homogeneizador Panda Plus 2000, Parma, Italy). After cell disruption and elimination of the insoluble fraction by centrifugation (23,700× *g*, 20 min, 4 °C) supernatants were heated at 70 °C for 30 min in order to denature thermolabile *E. coli* proteins, which were then discarded by centrifugation (23,700× *g*, 20 min, 4 °C). His-tagged proteins were purified by affinity chromatography on TALON CellThru resin columns following the manufacturer’s instructions (Clontech Laboratories, Inc., Palo Alto, CA, USA). Purified proteins were eluted in elution buffer (50 mM phosphate buffer pH 7.0, 300 mM NaCl, 150 mM imidazol) and further dialyzed and concentrated in 25–50 mM Tris-HCl buffer (pH 7.5) using Amicon Ultra concentrator tubes (30 kDa cutoff) (Millipore, Cork, Ireland). Proteins were visualized by SDS-PAGE, and concentrations were determined using the Bio-Rad Protein Assay (Bio-Rad, Hercules, California, USA) following the manufacturer’s instructions. Aliquots of the purified proteins were stored at −20 °C in 50% glycerol or kept without glycerol at 4 °C for immediate biochemical treatment in ATPase assays or transmission electron microscopy (TEM) preparations.

ATPase assays involved screening of ATP hydrolysis using the luciferin-luciferase ATP Bioluminescence Assay Kit CLS II (Roche Diagnostics, Mannheim, Germany), following the manufacturer’s indications. End-point kinetic assays were performed by employing different dilutions of the purified enzyme in ATPase activity buffer (5 mM MgSO_4_, 50 mM NaCl, 25 mM Tris-HCl pH 7.5, and 0.1 mM of ATP (Sigma-Aldrich, Saint Louis, MO, USA) for 1 h at 65 °C. At least four replicates per sample were conducted, including negative controls (protein with no substrate and spontaneous conversion control).

### 2.5. Single-Particle Electron Microscopy and Image Processing

Samples of purified HepA (2–5 mL at 0.3 mg/mL in Tris-ClH 20 mM pH 7.5) pre-incubated with 1–10 mM ATP (30 min, 65 °C with shaking) were applied to glow-discharged carbon-coated grids for 2 min. Grids were washed twice with water and negatively stained with 2% (w/v) aqueous uranyl acetate. Electron microscopy images were recorded on a CCD camera (4 k × 4 k resolution TEMCam-F416, TVIPS, Gauting, Germany) in a JEOL 1010 JEM electron microscope (JEOL, Tokyo, Japan) operating at 80 kV. Images were recorded at a 2.44 Å/pixel sampling rate, with an under-focus ranging from 0.7 to 1.5 μm.

General image processing operations were performed using Xmipp software [17] and graphics were produced by UCSF Chimera [18]. The CTF (contrast transfer function) was corrected with Ctffind3 [19] and images were down-sampled to a factor of 2, with a final sampling ratio of 4.88 Å/pixel. The Xmipp automatic picking routine was used to select 86,639 HerA particles. Images were classified using a reference-free clustering approach with the CL2D program [20] to select a homogeneous population of 86,506 particles. An artificial noise model was used as the starting reference for iterative angular refinement using the EMAN program [21]. The resulting model was selected and refined using the Xmipp iterative projection matching routine [22]. After independent refinement processes, 90% of particles were included in the final three-dimensional reconstruction with C6 symmetry, and the resolution of the model was determined by the Fourier shell correlation (FSC) criterion between independent half-dataset maps applying a correlation limit of 0.5.

The Chimera fitting routine was used to dock the HerA atomic model from *Sulfolobus solfataricus* [6] in the three-dimensional HepA map after initial manual placement. Each protomer of the crystallographic HerA hexamer was fitted in the cryo-EM map as an independent rigid body.

### 2.6. UV and Temperature Resistance Assays

Resistance against UV radiation and high temperature were measured as the ratio of viable cells after stress with respect to untreated controls. UV treatments involved a 60 min UV light exposure (Sylvania G8T5, Osram, Munich, Germany, λ = 254 nm, 10 cm) on liquid cultures of 0.5 mL extended on an empty Petri dish 3 cm in diameter. Thermal stress tests were carried out on unstirred cultures incubated for 18 h at 60 °C, 70 °C, and 75 °C. Tests were performed in triplicate and the strain used as the wild-type in these analyses was the *Δgdh::kat* mutant to discard any role of the Km resistance on the results.

### 2.7. Confocal Microscopy

Cells transformed with suicide plasmid pAB219 produce a HepA-sGFP fusion from its own promoter in the chromosome. For confocal microscopy analysis the cells were grown at 60 °C in TB medium to an OD_600 nm_ of 0.2 before a 4 mL aliquot was extended in a Petri dish (60 mm of diameter) and irradiated for 15 min with UV light (Sylvania G8T5, λ = 254 nm, 10 cm of distance). The culture was further allowed to grow at 60 °C for one more hour. When the HepA-sYFP was expressed ectopically from plasmid pAB175, the cells were subjected confocal microscopy in exponential phase (OD_600nm_ of 0.5). For confocal microscopy, around 5 × 10^8^ cells were washed once with PBS buffer [23], fixed for 5 min with 1.5% (w/v) paraformaldehyde, and after centrifugation and re-suspension in PBS, the cells were laid onto microscope slides previously covered with an ultra-thin 0.01% poly-L-Lysine and topped with Mowiol-treated cover slips. Images of cells were acquired with a LSM 710 confocal laser scanning microscope coupled to a vertical AxioImager M2 (Zeiss, Stockholm, Sweden) and an immersion objective 100X/1.4 oil Plan-Apochromat (Zeiss, Jena, Germany). All photographs were taken under the same settings of excitation/emission of a 514 nm argon laser. Bright field photos were also taken with Nomarski’s optics. Final image design was completed with Image J software (Wayne Rasband, NIH, Bethesda, MD, USA). Duplicates for every sample were prepared as well.

### 2.8. Transformation and Transjugation Assays

Quantitative transformation assays were performed as described [14] using exponential cultures grown at 60 °C and 15 or 150 ng of genomic or plasmid DNA, respectively. Transformation frequencies were expressed as the ratio of viable cells on selective plates versus viable cells in non-selective medium.

Transjugation experiments were conducted as described [14]. In short, matings involved mixing of the mates in the presence of DNase I (5 units; Roche) of 100 μL of saturated cultures of Hyg- and Km-resistant strains, previously washed in one volume of TB and then resuspended in 10 μL of TB containing DNase I (5 units; Roche) and plated onto sterile 0.22 μm nitrocellulose filters (GSWP, Millipore) on TB agar plates prior to incubation for 4 h at 60 °C. After incubation, cells were detached from the filters and resuspended in TB, and appropriate serial dilutions were plated onto selective agar plates. Transjugation frequencies were expressed as the number of CFU grown in double-selective media (Hyg + Km) per CFU grown on Hyg-containing agar plates. When matings involved chloramphenicol (Cm)-resistant wild-type strains the transjugation frequencies were referred to colonies grown on Cm-containing plates.

Statistical analysis of the transfer frequencies was performed using SPSS ^®^ Statistics v.21.0 (SPSS Inc., Chicago, IL, USA; 2008), considered statistically significant for *p*-values < 0.05. Inferential and comparative assays were performed when necessary and include Student’s *t*-tests, Kruskal-Wallis one-way analysis, Wilcoxon tests, and one-way analysis of variance (ANOVA) test, as described [15].

## 3. Results

### 3.1. Presence of HerA Homologs in Thermus

Among the members of the FtsK-HerA familiy of DNA translocases-helicases encoded by *Thermus thermophilus* HB27, the product of the gene of code TTC0147 shows the highest similarity to the HerA helicase of archaea [15]. HepA, the product of this gene, is 576 amino acids long, being conserved at the sequence level among all of the *Thermus* spp. so far sequenced (Supplementary Materials Figure S1). As in other members of this protein family, HepA includes an HAS barrel domain near its N-terminus and Walker A and Walker B motifs that suggest ATPase activity for this protein.

**Figure 1:**
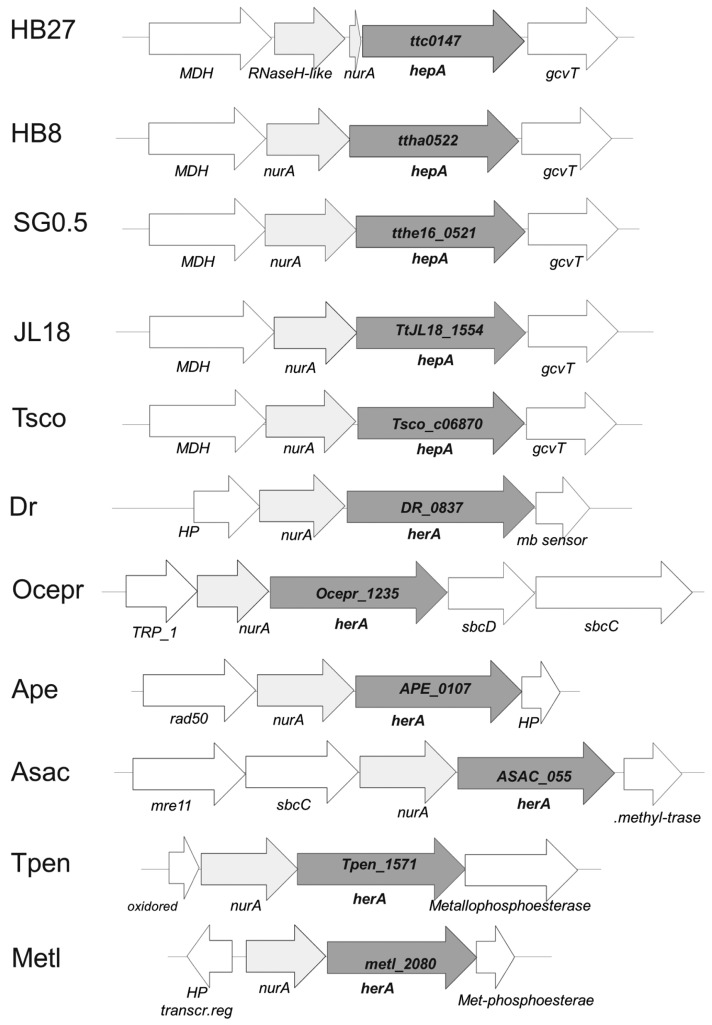
Genomic context of *hepA* homologs. Synteny of the cluster in which the common homolog of Hep*A* is found in *T. thermophilus* HB27 (*ttc0147*), *T. thermophilus* HB8 (*ttha0522*), *T. thermophilus* SG0.5JP16-17 (*tthe16_0521*), *T. thermophilus* JL18 (*TtJL18_1554*) and *T. scotoductus (Tsco_c06870)*, *Deinoccoccus radiodurans (DR_0837)*, *Oceanithermus profundus (Ocepr_1235)*, and the archaea *Aeropyrum pernix (APE_0107)*, *Acidolobus saccharovorans (ASAC_055)*, *Thermofilum pendens (Tpen_1571)*, and *Methanobacteirum lacus (metl_2080).*

The genome context of the *hepA* gene in different *Thermus* spp. is shown in Figure 1. As its archaeal HerA homologs, the *hepA* gene is preceded by a gene encoding a putative protein with sequence similarity to nucleases of the archaeal NurA family [24]. However, whereas in most *Thermus* spp. this NurA homolog is 293 amino acids long, in the HB27 strain the protein appears truncated, with its first 244 amino acids basically identical in sequence to its counterparts in other *Thermus* spp., and an unrelated eight amino acid extension, making a protein of 251 amino acids (NurAΔC). A detailed comparison revealed that this protein truncation is the consequence of a single nucleotide (cytosine) deletion at position 727 from the start codon of its coding gene. To be sure that this was not a consequence of an error in the published sequence, a whole genome re-sequencing of the HB27 strain was carried out, confirming the deletion in the *nurA*-like gene in two different stocks of the HB27 strain.

The gene context around the *nurA-hepA* genes has no apparent relation with DNA metabolism in *Thermus* spp. Actually, a homolog of the malic enzyme (NADP-dependent malate dehydrogenase) is encoded upstream from *nurA*, whose stop codon is separated only by 4 bp from the ATG start codon of *nurA*. In strains of *Thermus* spp. with a complete *nurA* gene, the stop codon of *nurA* overlaps the start codon of *hepA*. This suggests the existence of co-transcription of the malic enzyme gene and the *nurA-hepA* tandem. On the other hand, 13 bp downstream from *hepA* a putative glycine cleavage system aminomethyltransferase T (gcvT) is encoded, which is followed by other genes apparently related to glycine metabolism. This genomic structure is also conserved in other *Thermus* spp. (Figure 1), supporting that the *nurA-hepA* gene duet has been acquired in an ancestor of this genus. In agreement to this, the G + C content of the *nurA-hepA* genes seems homogeneous with respect to that of the genome average (69%). In contrast, in *Oceanithermus profundus*, another genus of the *Thermales* order, genes encoding homologues to the bacterial SbcD and SbcC proteins, related to archaeal Mre11 and Rad50 proteins, are encoded downstream of *hep*A, supporting for this organisms a gene architecture and function similar to that shown by the DSB repair system of archaea. Having in mind the higher similarity in protein sequence between the HepA proteins of *T. thermophilus* and *O. profundus* (66% of identity), compared to that of each of these proteins and any archaeal protein of the HerA family so far sequenced (*Sulfolobus islandicus* WP_014512739.1 showing the highest identity at 27%), these data suggest the acquisition of the *hepA* gene by an ancestor common to both genera followed by the recruitment of SbcD and SbcC proteins in *O. profundus*.

### 3.2. Localization of HepA

**Figure 2:**
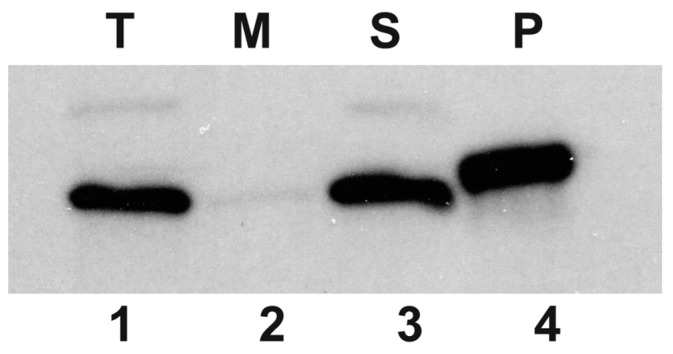
HepA is a cytoplasmic protein. Western blot with an anti-HepA antiserum on whole cells of exponential culture of *T. thermophilus HB8* (T, lane 1), and its soluble (S, lane 3) and membrane fractions (M, lane 2). Purified His-tagged HepA was employed as the control (P, lane 4).

In order to associate the HepA protein to a cell fraction, a polyclonal rabbit antiserum was raised against this protein that allowed its identification by Western blot. As shown in Figure 2, a protein of the expected size for HepA (64 kDa) was identified in the soluble fraction, whereas this protein was not detected in the insoluble membranes and cell wall fraction. These data show that HepA is produced as a soluble cytoplasmic protein, in agreement with bioinformatic predictions.

**Figure 3:**
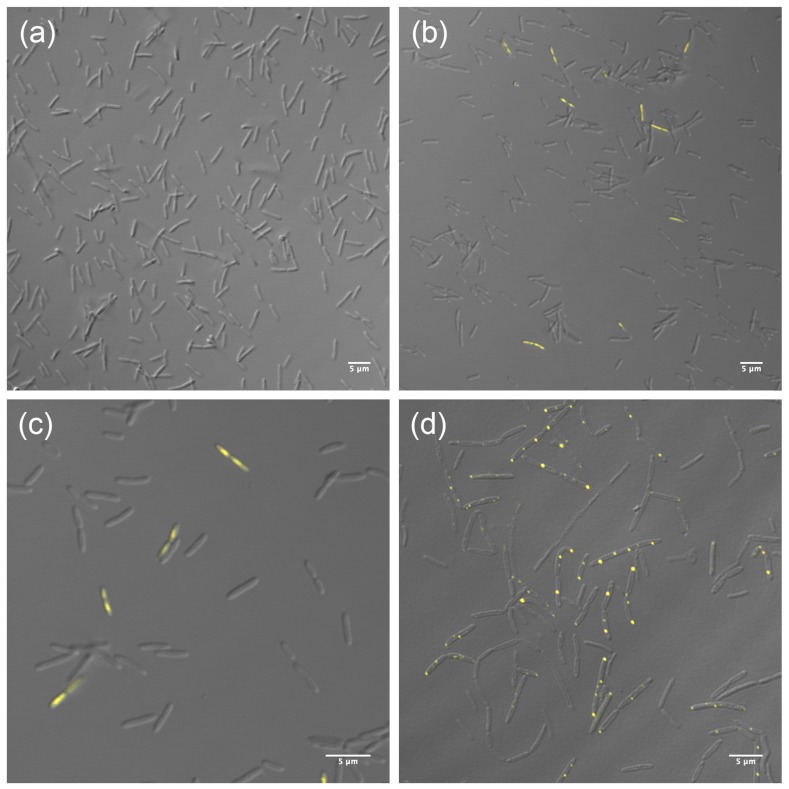
Localization of HepA. HepA-sYFP fusion expressed from a single copy in the chromosome under its natural promoter (**a**–**c**) or ectopically from a multicopy plasmid in a constitutive way (**d**). Panel (**a**) corresponds to untreated cells. Panels (**b**) and (**c**) correspond to different magnifications of UV-treated cells as described in the Materials and Methods Section.

To further localize the protein within the cells, a fusion of HepA to a thermostable fluorescent protein (sYFP) was obtained and expressed both, as a single copy, from its own promoter in the chromosome, and also ectopically from a constitutive promoter in a multicopy plasmid. The expression of the HepA-sYFP fusion from a single copy did not allow the detection of significant fluorescent signals over the background in untreated cells (Figure 3a). However, when these cells were treated with UV (see the Materials and Methods Section) and further allowed to grow for one hour at 60 °C, fluorescent signals were detected in a percentage of the cells (10%, approximately) (Figure 3b). In these, the fluorescence was extended over the whole cell, likely associated to the nucleoid (Figure 3c). On the other hand, when expressed ectopically from a plasmid, the fluorescence accumulated as very intense dots, most of them of polar localization (Figure 3d). These data support, first, that the protein is expressed at low levels in normal cells and, second, that upon stress the protein is induced by a percentage of the population. Overexpression of the protein fusion from a plasmid could represent a non-physiological situation.

### 3.3. The Role of HepA

**Figure 4:**
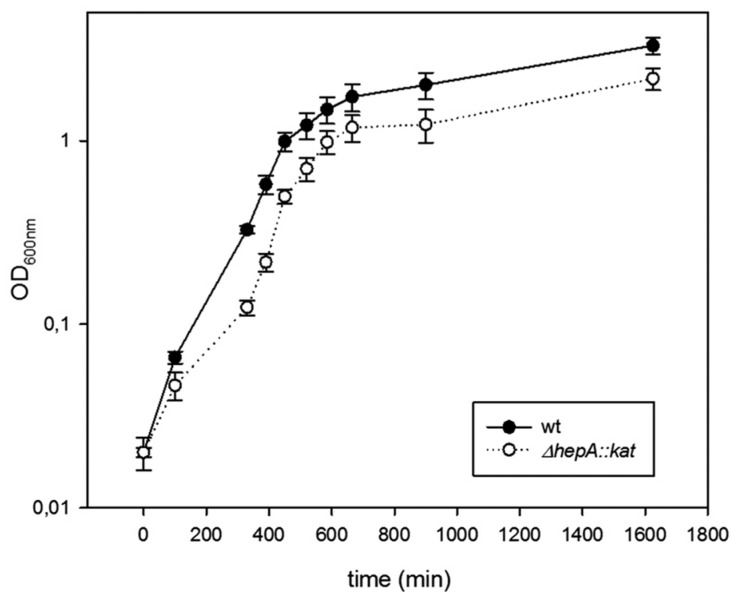
Mutants defective in HepA grow at low temperatures. Growth curves in TB medium at 60 °C of the wild-type strain (black circles), and its *ΔhepA* mutant (empty circle). Error bars represent the deviation of the mean value of three independent samples.

The role of HepA in the physiology of *T. thermophilus* was investigated through the behavior of deletion mutants. As shown in Figure 4, the Δ*hepA::kat* mutant grows at 60 °C with similar rates compared to a *gdh::kat* mutant used as a wild-type control, but with a small delay in growth initiation. Final cell yields are also similar in both strains under these conditions.

In order to know if the HepA protein was required for growth at higher temperatures, parallel growth assays starting with the same number of cells from inocula grown at 60 °C were carried out at 60 °C, 70 °C, and 75 °C with the same two strains, and viable cells were counted after 18 h of incubation. As shown in Table 4, when the ratio of viable cells for the Δ*hepA::kat* vs. that of the *gdh::kat* mutants were compared, similar growth was detected for both strains at 60 °C (ratio = 1.1 ± 0.05), as expected from our data of Figure 4. However, this ratio decreased dramatically with the increase in growth temperature, in such a way that it was less than 1:10 at 70 °C and 1:1000 at 75 °C. This means that HepA is required for efficient growth at high temperatures in *Thermus thermophilus*.

**Figure 5:**
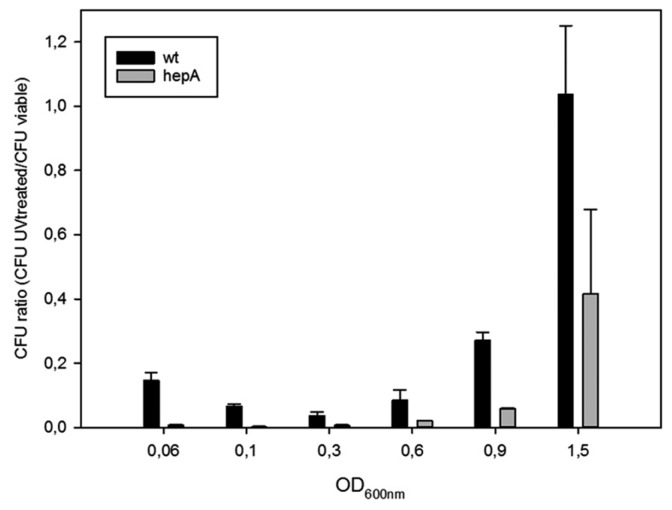
Sensitivity of *hepA* mutants to UV. Figure shows the ratios between viable cells of UV treated and untreated cells at different cell densities (OD_600_) along the growth curves of *T. thermophilus* HB27 *gdh:.kat* (wt) and *T. thermophilus* HB27 *ΔhepA::kat* mutant.

The role of HepA in resistance to UV was also studied. For this, the same pair of mutants was treated with UV at different points of their growth curves, and the ratios between viable cells after and before the UV treatment were measured. As shown in Figure 5, wild-type cells (actually the *gdh::kat* mutant) are much more sensitive during exponential growth than cells reaching the stationary phase, in such a way that ratios close to 1:100 were found at late exponential growth, and the resistance increases to almost complete resistance when the cells reached OD_600_ around 1.5. In the *hepA* mutant a similar pattern was detected, but showing a much higher sensitivity, in such a way that even cells at OD_600_ of 1.5 the cells showed a dramatic sensitivity to UV treatment. These data support that HepA is involved in DNA repair processes.

### 3.4. HepA is Required for DNA Donation in Transjugation

**Figure 6:**
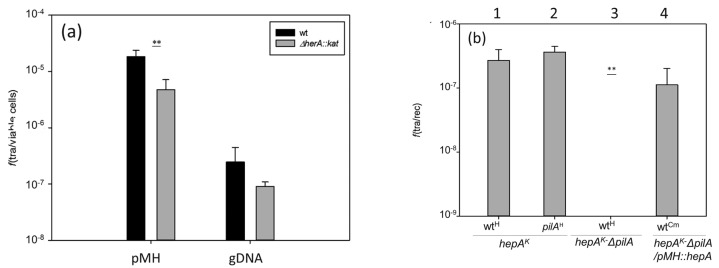
Effects of the absence of HepA in transformation and transjugation. (**a**) Parallel cultures of *T. thermophilus* HB27 *gdh::kat* (wt, black bars) and the *ΔhepA::kat* mutant (gray bars) were transformed with 150 ng of plasmid pMH118 or with 15 ng of genomic DNA from an isogenic strain labelled with Hyg resistance in the chromosome; and (**b**) transjugation assays between the indicated mates. (1) *ΔhepA::kat* × wild-type*::hyg*; (2) *ΔhepA::kat* × *ΔpilA::hyg*; (3) *ΔhepA::kat-ΔpilA* double mutant × wild-type*::hyg;* (4) *ΔhepA::kat-ΔpilA* double mutant containing a plasmid expressing HepA ectopically × wild-type resistant to Cm. (*p*-value < 0.005 is shown as **).

*Thermus thermophilus* shows a high capability for horizontal gene transfer due to the concomitant and overlapping concurrence of natural competence and a cell-to-cell transfer mechanism called transjugation [15]. The influence of the HepA protein in these two processes was investigated. As shown in Figure 6a, in the absence of HepA, a significant reduction (around 10-fold) in transformation efficiency was detected with both plasmid and genomic DNA. To analyze its putative relevance in transjugation, and having in mind that in this process both mates can act as donors and recipients (bidirectional) [14], we used a double mutant also affected in *pilA*, a mutation that makes the cells incapable to act as recipients in the process, but that does not affect its capability for DNA donation [14]. As shown in Figure 6b, crosses between the *hepA* mutant and a wild-type strain, or even a *pilA* mutant, produced transjugant colonies (bars numbered 1 and 2). By contrast, the double *hepA-pilA* mutant did not produce transjugants in mating with a wild-type strain (bar number 3), supporting that the *hepA* mutants are affected by DNA donation. This defect in DNA donation was rescued through the ectopic expression of HepA from a plasmid (bar number 4).

### 3.5. HepA Shows Low ATPase Activity

**Figure 7:**
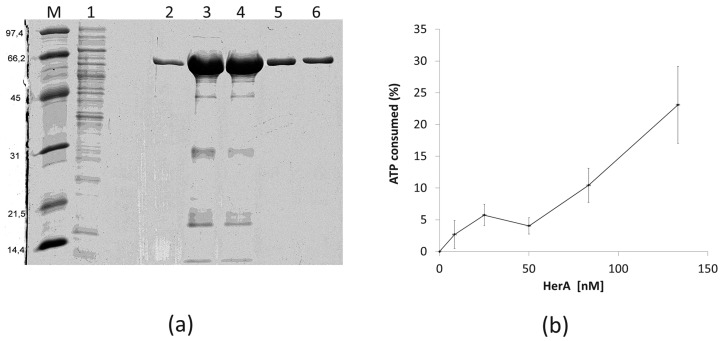
Purification and ATPase activity of HepA. (**a**) Coomassie blue-stained SDS-PAGE gel showing purification of HepA as described in materials and methods. (M) Proteins of the indicated size (kDa) used as markers; (1) IMAC column flow-through; (2–6) fractions eluted with imidazole. (**b**) ATPase activity expressed as the ATP consumed (%) after incubation for 1 h at 65 °C with the indicated concentrations of HepA. The initial concentration of ATP was 10^−4^ M.

In order to know if the HepA protein has similar biochemical and structural properties as its archaeal HerA homologs, we purified a recombinant His-tagged protein and assayed its ATPase capability. As shown in Figure 7a, a protein of the expected size was overexpressed in *E. coli* and purified by affinity chromatography from the soluble fraction. The protein was further shown to have ATPase consumption capability at 65 °C in a protein concentration-dependent manner (Figure 7b), although the activity detected was low, with only a 25% of the ATP consumed after 1 h (around 200 nM of ATP per nM of HepA protein).

### 3.6. HepA Is a Complex Hexameric ATPase

**Figure 8:**
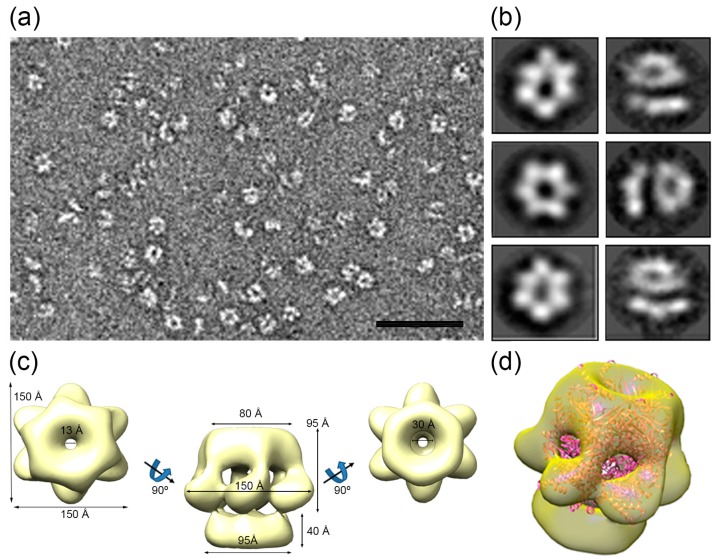
HepA single-particle electron microscopy reconstruction. (**a**) Representative electron micrograph of a negatively-stained HepA sample. Bar, 50 nm; (**b**) six two-dimensional averaged classes of the oligomeric HepA; (**c**) three-dimensional reconstruction of the hexameric HepA with C6 symmetry; and (**d**) a semitransparent model of the hexameric HepA with a docked atomic model of the hexameric HerA (pink).

Purified HepA assemblies obtained after incubation with ATP were analyzed by negative stain electron microscopy (Figure 8a). Two-dimensional classification of the images showed that approximately half of the two-dimensional averaged images showed six-fold symmetry (Figure 8b, left), and 86,506 images were used to generate a three-dimensional reconstruction of the hexameric HepA. The final resolution of the model was estimated to be 16 Å. The HepA oligomeric assembly is a complex hexameric ring with two components, a 95 Å-long ring with external diameters of 80 and 150 Å on each side (the so-called upper ring), connected by the wider side to a flat ring (the so-called lower ring), 37 Å long with an external diameter of 95 Å (Figure 8c). The upper ring has six 25 × 15 Å diameter holes on the side. The HepA internal channel has a diameter of 13 and 30 Å at its ends, suggesting that conformational changes are necessary to allow dsDNA passage.

We compared the HepA map with structural homologues, such as the hexameric HerA X-ray structure from *S. solfataricus* [6] (Figure 8d). Whereas the HerA model (Protein Data Bank (PDB) ID 4D2I) fits well within the upper ring, the lower HepA ring is empty, suggesting major structural differences between HerA and HepA.

## 4. Discussion

Several genes from archaeal origin have been identified in thermophilic bacteria that might be adaptive elements to high temperatures [3], but little experimental data supports such hypothesis. One of these putative adaptive genes from archaeal origin is *hepA*, encoding a homolog of the HerA, an archaeal helicase, which plays a major role in DSB repair through helping in homologous recombination [25,26]. Here we show that the product of *hepA* is actually a hexameric ATPase required for growth at high temperatures and also for survival to DNA damage. However, a number of questions remain unanswered, especially regarding the actual activity of the HepA protein on DNA metabolism and the possible requirement for a nuclease of the NurA family to enhance its activity.

The EM image reconstruction of HepA reveals the classic structure for DNA translocases and helicases, with a central channel of an apparent size of 13 Å which, theoretically, could only accommodate single-stranded DNA. However, in a recent article describing the X-ray structure of HerA from *S. solfataricus* in complex with DNA, it was shown that the HerA helicase accommodates up to three helix turns of B-dsDNA through a central channel 25 Å wide, pushing it in a mechanism likely dependent of ATP hydrolysis [6]. The fitting of the HerA structure in the EM HepA map indicates that the relatively small size of the central channel in the structure (to passage of dsDNA) was either the consequence of drying and/or negative staining or, alternatively, it could represent an inactive form of the enzyme. Actually, our ATPase activity assays are in agreement with this later hypothesis, as the catalytic activity detected for the hydrolysis of ATP by HepA was very low (0.05 s^−1^), despite incubation at 65 °C with this substrate was required for the formation of the hexameric rings.

Our HerA docked model in the HepA map also shows HepA regions at the bottom anneal of the structure that the HerA atomic model lacks (although the last 23 residues at the C-terminal were disordered and did not appear in the HerA structure [6]). Considering that the hexameric HepA structure shows larger cross-section dimensions (50%) than that described for the HerA crystal, and that both proteins contain a similar number of amino acid residues, it is tempting to speculate that the compacting level of HepA is much lower than that of HerA. Higher resolution analysis will be required in the future to confirm this working hypothesis.

As it happens with HepA, the HerA protein of *S. solfataricus*, and also that of its homolog in *D. radiodurans*, shows very low stand-alone ATPase activity. However, this low activity is greatly stimulated by direct interaction with the corresponding nucleases of the NurA family and/or DNA [7] which, for HerA, seems essential in archaea [27]. In all the genomes of *Thermus* spp. so far available, the HerA homologs (HepA proteins) are preceded by a NurA homolog, supporting that the NurA-HepA tandem is also the active form in *Thermus* spp. However, in *T. thermophilus* HB27, the NurA homolog gene preceding *hepA* appears mutated as a pseudogen [28]. Therefore, either the missing C-terminal domain, absent from this NurAΔC truncated form, is not required for the formation and activity of the NurA-HepA complex, or another NurA homolog substitute for it in this strain. Actually, the HB27 strain contains additional proteins of the NurA family that are absent from its close relative *T. thermophilus* HB8 [15]. Therefore, the possibility exists of functional replacement of this NurAΔC truncated form by another NurA-like protein specific to this strain. In fact, a mutant in this second NurA homolog (code TTC_1878) was as defective in transjugation [15] as the *hepA* mutant.

Regarding the physiological role played by HepA in *T. thermophilus*, our data show a significant role in survival to UV treatments, likely acting in a homologous recombination-dependent repair pathway, as described for HerA. Two arguments are in favor of this. First, the protein seems to be overproduced at least by a significant fraction of cells following UV treatment (Figure 3b). Second, the protective role of HepA seems more relevant in cells growing exponentially (Figure 5), where the higher copy number of the chromosome makes homologous recombination more relevant as a repair mechanism than in the stationary phase, where single, or a much limited copy number of the chromosome, are present.

The dramatic effect of the absence of HepA in the ability of the cells to grow at high temperature was unexpected, having in mind that growth at 60 °C was similar to that of a wild-type strain (also labeled with a Km marker for comparison). Actually, these results were somehow similar to that shown for *recA* mutants of *T. thermophilus*, where growth above 60 °C severely affected the viability and, especially, the fidelity of replication, leading to some class of catastrophic mutagenesis in the cells [29]. The possibility exists that growth at high temperatures could increase the chances for DSB during replication and that the role of HepA could, in this sense, be related to that of HerA. However, the requirement for HepA in additional unknown DNA repair mechanisms cannot be discarded.

Regarding the putative role of HepA in transformation, it seems clear that integration of a genomic marker when the incoming t-DNA is lineal depends on double homologous recombination with the chromosome, and in this context a decrease of about one order of magnitude in transformation efficiency of the *hepA* mutant with respect to a wild-type is also in agreement with a role for HepA in the recombination process. More difficult to explain is the role of HepA in transformation with a replicative plasmid, for which a decrease of an order of magnitude in efficiency was also observed in the *hepA* mutant. It could be speculated to be a requirement of recombination during the transformation process, even with a plasmid, but this has not been previously described. Alternatively, the transformation process could be defective just because the bacterium has difficulties for growth or because plasmid replication is somehow affected. Whatever the explanation of this defective transformation could be, no supportive data exist to favor any of them.

Transjugation is a process recently described in which a donor *T. thermophilus* cell donates DNA to a recipient cell which actively import the DNA through its competence apparatus [15]. In this process, the HepA protein seems to be an absolute requirement for DNA donation, as a mutant defective in *pilA* (unable to act as a recipient) in combination with a *hepA* mutation, cannot donate DNA to a wild-type strain (Figure 6). The transjugation process, itself, is actually a class of generalized conjugation as it starts simultaneously at several points in the chromosome of the donor cell [14]. We speculate that acting as a DNA donor requires multiple cuts along its chromosome to generate DNA fragments appropriate for donation. The presence of multiple copies of the chromosome [30] would allow the donor to maintain its genetic stability by homologous recombination-mediated DNA repair, a process in which the HepA protein could be an absolute requirement.

Independently of the actual molecular mechanism of DNA repair in which HepA could be involved, the fact is that its absence makes the cells more sensitive to UV and limits the growth temperature at which the strain can grow, supporting that its acquisition from a thermophilic archaea could have helped this genus to adapt to new high-temperature environments. In this scenario, it is of note that the clusters of genes in which the *nurA-hepA* duet is integrated is fully conserved in all of the genomes of *Thermus* spp. so far analyzed, and different from that of its clade mate genus *Deinococcus*. Therefore, our data point to independent acquisition of this *nurA-hepA* gene tandem in both genus.

## 5. Conclusions

Transfer of DNA has been detected in all three domains of life, making an intense impact in the roots of the phylogenetic tree, grounded among thermophiles. Indeed, comparative analysis of whole genome sequences have unveiled that many genes among thermophilic genomes have been acquired by HGT from external sources, principally thermophilic Archaea, likely helping these organisms to adapt to extreme temperatures. One of these genes of archaeal origin that have likely contributed to thermal adaptation upon acquisition by an ancestor of the genus *Thermus* is *herA*, which in Atchaea encodes a helicase implicated in double strand DNA breaks repair mechanism involving recombination. The role of its homologue in *Thermus* spp., named as HepA, has been analysed showing to be an hexameric cup-like structure with low ATPase activity. Its mutation produces a phase-growth dependent temperature and UV sensitive phenotype, compatible with a role in DNA repair required for high temperature growth. Further studies are needed to examine the complex NurA-HerA in DNA repair systems and DNA transfer.

## Figures and Tables

**Table 1:** Strains used in this work.

Strain	Genotype	Source
*E. coli* DH5α	*supE44 ΔlacU169 (Φ80 lacZΔM15) hsdR17*, *recA1*, *endA1*, *gyrA96*, *thi-1 relA1*	[10]
*E. coli* BL21 (DE3)	*F^-^ ompT gal dcm lon* HsdSB (r_B_^-^m_B_^-^) λ(DE3 [*lacI lacUV5-T7* gene 1 *ind1 sam7 nin5*])	[11]
*T. thermophilus* HB27	*ATCC BAA-163/DSM7039*	Y. Koyama
*T. thermophilus* NAR1	*[pTT27::nar]*	[12]
*T. thermophilus* HB8	*ATCC 27634*	Y. Koyama
*T. thermophilus* HB27^EC^	*HB27 ago::agoISTth7*	[13]
*T. thermophilus* HB27^Cm^		This work
*T. thermophilus* ∆*pilA4*	HB27^EC^ *∆pilA4*	[14]
*T. thermophilus* HB27 ∆*pilA4::hyg*	HB27^EC^ *ΔTTC0858::hyg*	[14]
*T. thermophilus* HB27^H^	HB27^EC^*ΔTTC0313::hyg*	[14]
*T. thermophilus* HB27 *gdh::kat*	HB27 [∆*TTC1211::kat*]	[12]
*T. thermophilus* HB27 *hepA:*:pk	HB27^EC^ *TTC0147::pK18*	This work
*T. thermophilus* HB27 *∆hepA::kat*	HB27^EC^ ∆*TTC0147::kat*	This work
*T. thermophilus* HB27 *∆hepA::hyg*	HB27^EC^ ∆*TTC0147::hyg,*	This work
*T. thermophilus* HB27 *∆hepA::kat pilA4*	HB27^EC^ ∆*TTC0147::kat*, *∆pilA4*	This work
*T. thermophilus* HB27 *hepA*YFPph	HB27^EC^ [*TTC0147-sYFP:pH118]*	This work
*T. thermophilus* HB27 *∆hepA, ∆pilA4* PMH	HB27^EC^ ∆*TTC0147::kat*, *∆pilA4* [pMH::*TTC0147*]	This work

**Table 2:** Oligonucleotides used in this work. In capital letters, the annealing sequence and, underlined, the cloning restriction site employed.

Primer (Use)	Sequence 5′–> 3′
TTC0313dir	CTTTACGAGGCCCTCTTGGAG
TTC0313rev	CCACCGCTCGGGGAC
AB92 (check *pilA4* deletion)	AAATGCTGAAGCTTGGCGGCAAC
AB93 (check *pilA4* deletion)	AAAAGAATTCGGGAGTTAGGCTTGGGATTGTG
AB219 (check *hepA* deletion)	CTACCTGAAGAACTCCCGGCGCAG
AB220 (check *hepA* deletion)	GTGAAGCGTATCGGCGTGGTCTTG
AB221 (insertion *hepA*)	aaagaattcGAGGTGGCCTACCTCAACCTG
AB222 (insertion *hepA*)	aaactgcagGAGCTCGTCCAGGACGATGAAG
AB231 (*hepA*-YFP fusion)	actagtCCTGAAGAACTCCCGGCGCAG
AB232 (*hepA*-YFP fusion)	ccatggGTGAAGCGTATCGGCGTG
AB249 (deletion *hepA*)	aaagaattcGTAATCTAGGGGCATGGCCTG
AB250 (deletion *hepA*)	aaatctagaCTTCACCTCGCACCTCCCA
AB251 (deletion *hepA*)	aaatctagaGATGGCCTCGCCATGAAGA
AB252 (deletion *hepA*)	aaactgcagCAGGGGCGAATAC
AB247 (overexpression *hepA*)	aaacatatgGTGAAGCGTATCGGCGTG
AB248 (overexpression *hepA*)	aaaaagcttCTACCCGAAGAACTCCC

**Table 3:** Plasmids employed in this work.

Plasmid	Description/Use	Reference
pET28b(+)	Recombinant expression of proteins in *E. coli*	Novagen
pUC19/18	Cloning vector	[16]
pUC19*::kat*	pUC19 with the thermostable kat resistance gene cassette	This work
pUC19*::hyg*	pUC19 with the Hyg resistance gene cassette	This work
pK118	Suicide vector for *T. thermophilus*, Thermostable Km resistance	[12]
pH118	Suicide vector for *T. thermophilus*, Thermostable Hyg resistance	[15]
pMK184	Cloning vector for *T. thermophilus.* Km resistance	[15]
pMH184	Cloning vector for *T. thermophilus.* Hyg resistance	[15]
pMHPnqosYFP	Expression of sYFP in *T. thermophilus*	[12]
pAB135	pET28b derivative for overexpression of N-terminal His-tagged HepA	This work
pAB207	pUC18::*hepA::kat*. For the isolationon of Δ*hepA::kat* mutants	This work
pAB219	pH118:*hepA*::sYFP. pH118 derivative to generate chromosomal fusions of HepA to sYFP	This work
pAB175	Derivative of pMHPnqosYFP for the ectopic expression of the HepA-sYFP fusion in *T. thermophilus*	This work
pAB150	Plasmid for the isolation of Hyg resistant single insertion *hepA* mutants	This work
pAB151	Plasmid for the isolation of Km resistant single insertion *hepA* mutants	This work
pAB141	Hyg-resistant plasmid for the *hepA* mutants	This work

**Table 4:** Sensitivity to temperature of *hepA* mutants.

Viable CFUs Ratio	60 °C	70 °C	75 °C
wt (gdh)	18.7 ± 1.7 × 10^7^	40.9 ± 8 × 10^7^	16.30 ± 1 × 10^7^
*ΔhepA*	20.1 ± 9.3 × 10^7^	33.3 ± 1 × 10^6^	12.8 ± 3.9 × 10^4^
*ΔhepA*/wt	1.1 ± 0.05	8.16 ± 1.3 × 10^−2^	7.8 ± 0.004 × 10^−4^

## Supplementary Materials

The following are available online at www.mdpi.com/2073-4425/8/5/130/s1. Figure S1: Sequence alignments of HepA homologs.
