# Supplementary material for: Role of Archaeal HerA Protein in the Biology of the Bacterium Thermus thermophilus
Source: Genes (Basel). 2017 Apr 27;8(5):130. doi: 10.3390/genes8050130 (PMC5448004; doi:10.3390/genes8050130)
Supplement: Supplementary file 1 [file genes-08-00130-s001.pdf]

**Figure S1.** Role of archaeal HerA protein in the biology of the bacterium *Thermus thermophiles* (Blesa et al, 2017).

```
Deinococcus          MTGNDVQGAEKADAIGMVLGTEDVTPTVFWFAVSHGASVGLDDLVVVETRKPD--GTPVR
Ocep                 ---------MKQEAIGVVLGSREASPLEFWIGIQ-DGLVRLDDVVWVEADHGG--L-EVR
Tsco                 -----------MERIGVVLGRREATPLEFWVGVEGEGLLRLDDLVVVEGFHPK--VGKVR
JL18                 -----------MKRIGVVLGRREATPLEFWVGVEGDGLLRLDDLVVVEGFHPQ--VGQVR
HB27                 -----------MKRIGVVLGRREATPLEFWVGVEGDGLLRLDDLVVVEGFHPQ--VGQVR
HB8                  -----------MKRIGVVLGRREATPLEFWVGVEGDGLLRLDDLVVVEGFHPQ--VGQVR
SG0.5                -----------MKRIGVVLGRREATPLEFWVGVEGDGLLRLDDLVVVEGFHPQ--VGQVR
Sulfolobus           ------------MIIGYVVGSATTQEANVLLEK----KVRSGYYVTLE-YDDEKVLGLVT
Archaeoglobus        -----------MQNVGLVMGKSSITDFSFAVNPSA--IPKFGEYVTAINRDGEEVIGIVR
Methanocaldococcus   ------MMVINSVVVGTVVASKNVNEFEFVIENQVIDKIKKGEFVITKNTHGDYLLSKIT
                                    :* *:.      . .       .  *          :

Deinococcus          FYGLVDNVRKRHEGVTFESDVEDVVAG---------LLPASVSYAARVLV-----TRVDP
Ocep                 YYGMVDRVHKVLEGTQFDSDTFLAAGN---------LIPTNTAYVAHVTV-----TRLEP
Tsco                 YFGMVDLVAKAHEGESFDTDVFLAVQG---------KIPVSLAYVAHVSV-----TRIVP
JL18                 FFGMVDHVAKVHEGESFDTDTFLAVEG---------KLPVSLAYVAHVSV-----TRILP
HB27                 FFGMVDHVAKVHEGESFDTDTFLAVEG---------KIPVSLAYVAHVSV-----TRILP
HB8                  FFGMVDHVAKVHEGESFDTDTFLAVEG---------KIPVSLAYVAHVSV-----TRILP
SG0.5                FFGMVDHVAKVHEGESFDTDTFLAVEG---------KLPVSLAYVAHVSV-----TRILP
Sulfolobus           ---LITTGSPLVDDSLNDIELVQRIKQ------MGNKIPIYMKAKVKLLCKLD--GKLSQ
Archaeoglobus        ---EISNFNRMILDEGFSFEYL--VKNLDFSRRLIEKNDVIVATATVIGVVKD--GEVYP
Methanocaldococcus   ---KIVSVNALIGDKSEDASELAKIRGVIYSEEMLNNSSKFLASAKILGVINNESGSIES
                       :       .   . .                        :        :

Deinococcus          ENFIPPQPGDHVRHAAGRELAMALSADKMEE----AAFPGGLLADGQPLPLNFRFINGES
Ocep                 EEYLPPDPGSPVYLARQEALENALYYDRMKQ-----KLPVGFMRNGEPAYVNLAFLDGRQ
Tsco                 EEFFPPDPGSAVYLARGEDLELALYYDAMKNQRGSTKLPAGFLKSGEVAYLNLEFLNGVK
JL18                 EEFFPPDPGSPVYLAQEEDLELALYYDAMRNQRGSTKLPAGLLKNGEVAYLNLEFLNGVK
HB27                 EEFFPPDPGSPVYLAQEEDLELALYYDAMRNQWGSTKLPAGLLKNGEVAYLNLEFLNGVK
HB8                  EEFFPPDPGSPVYLAQEEDLELALYYDAMRNQRGSTKLPAGLLKNGEVAYLNLEFLNGVK
SG0.5                EEFFPPDPGSPVYLAQEEDLELALYYDAMRNQRGSTKLPAGLLKNGEVAYLNLEFLNGVK
Sulfolobus           PDL-PPVAGTPVRLATNEELSTIF-SEG--------TIRIGKLI-GSDVEVRIRVNALTR
Archaeoglobus        NRT-PIKPNSEVFLADDDVLSSLFKCDR--------GVELGRMIARPDISVSLDIKQLVL
Methanocaldococcus   NVY-PINVPQNVYLTKDDLLAKIF-SNG--------SIEVGYLKVRSSTKVKLNAKELCS
                       *    * :   *  :  :        .  * :      : :

Deinococcus          GGHINISGISGVATKTSYALFLLHSIFRSGVMDRTAQGSGGRQSGTAGGRALIFNVKGED
Ocep                 GGHVNISGISGVAAKTSYALFLLHSLYNSGVLEDAAS-----------AKTLIFNVKGQD
Tsco                 GGHVNISGISGVAAKTSYATFLLKSLLESGVLEEAHQ-----------ARVLLFNVKGED
JL18                 GGHVNISGISGVAAKTSYATFLLKSLLESGVLEDAHQ-----------AKVLLFNVKGED
HB27                 GGHVNISGISGVAAKTSYATFLLKSLLESGVLEDAHQ-----------ARVLLFNVKGED
HB8                  GGHVNISGISGVAAKTSYATFLLKSLLESGVLEDAHQ-----------ARVLLFNVKGED
SG0.5                GGHVNISGISGVAAKTSYATFLLKSLLESGVLEDAHQ-----------ARVLLFNVKGED
Sulfolobus           --HLAILAATG-SGKSNTVAVLSSRLSE----VFG--------------SVLIFDYHGEY
Archaeoglobus        R-HFAILSVTG-GGKSNTVAVLVNDIVK----KLNG-------------TVVLIDPHGEY
Methanocaldococcus   R-HFAVLAMTG-AGKSNTIAVLVQELFE----KDKGK-----------MNIVIVDPHGEY
                      *. : . :* . *:.   .*    :                      ::.: :*:
```

```
Deinococcus          L-LFLDKPNARMVEKEDKVVRAKGLSADRYALLGLPAEPFRDVQLLAPPRAGAAGTAIVP
Ocep                 L-FYLDKPNRELNEKERE----------RYRRLGLEPEPFSSVAFFAPPRLA-QGQGHLV
Tsco                 L-LFLDKPNLRLSEEAKE----------EYRRLGLPPTPFGSVRFLAPPKKGEEGI--LP
JL18                 L-FFLDKPNARLTEEARK----------AYARLGLPATPFQSVAFLAPPKKAGY----LP
HB27                 L-FFLDKPNARLTEEARK----------AYARLGLPATPFQSVAFLAPPKKAGY----LP
HB8                  L-FFLDKPNARLTEEARK----------AYARLGLPATPFQSVAFLAPPKKAGY----LP
SG0.5                L-FFLDKPNARLTEEARK----------AYARLGLPATPFQSVAFLAPPKKAGY----LP
Sulfolobus           Y-----ESEIKNLNNIEP----------KINPLNLTPDEFAT----------------LL
Archaeoglobus        VGYTFEDGSERGKNVVPA----------GIRPERLEPWEFAS----------------LV
Methanocaldococcus   V-------KMRNTHILPA----------KLNPILVPEEHLAK----------------LL
                            .                          :    :                  :

Deinococcus          QTDQRSEGVTPFVFTIREFCARRMLPYVFSDASASLNLGFVIGNIEEKLFRLAAAQTGKG
Ocep                 AASSRAEGVRPYHWSIVDFCQDGLLPFLFTDRGAMSNLGFLIDHVTARLAALARGQ--EG
Tsco                 DVETRLEGVKAYHWDLVQFAQKGLLPFLFTDKGALTNLGFLVAHVTEKLRRLAEGQ--KG
JL18                 DVDTRLEGVEAYHWDLVQFCQRGLLPFLFADRAAMTNLGFLVAHVTEKLKRLAEGQ--KG
HB27                 DVDTRLEGVEAYHWDLVQFCQRGLLPFLFADRAAMTNLGFLVAHVTEKLKRLAEGQ--KG
HB8                  DVDTRLEGVEAYHWDLVQFCQRGLLPFLFADRAAMTNLGFLVAHVTEKLKRLAEGQ--KG
SG0.5                DVDTRLEGVEAYHWDLVQFCQRGLLPFLFADRAAMTNLGFLVAHVTEKLKRLAEGQ--KG
Sulfolobus           EI-RENATIQ------YRILRRAFKSFLEETK----------------------------
Archaeoglobus        GVDREKAAVQ------RMHLERIFTTVRHEGKAG-----------REFVERVLDIA----
Methanocaldococcus   GIG-ENSSVQ------KSFLVYAALTVKYECKENKKQISGL-EYLKKIEEKLVECA----
                            :                 .

Deinococcus          TGLIVHDWQFEDSETPPENLDFSELGGVNLQTFEQLISYLEYKLLEEREGE-----GDPK
Ocep                 PELYVDDWPEGPTGEA---GAFDSLGRVKIGSFGQLVDYLEFQLLGAES-EGEAASGNHH
Tsco                 PHLLVADWPGGELPED---ITFDDLGRVRLKSFADLVRYLEYKLLGPETGEGE---GDRT
JL18                 PHLQVEDWPGGEASEG---MSFDQLGKARLKSFSDLVRYLEYKLLGPETGEGE---GDRG
HB27                 PHLQVEDWPGGEASEG---MSFDQLGKARLKSFSDLVRYLEYKLLGPETGEGE---GDRG
HB8                  PHLQVEDWPGGEASEG---MSFDQLGKARLKSFSDLVRYLEYKLLGPETGEGE---GDRG
SG0.5                PHLQVEDWPGGEASEG---MSFDQLGKARLKSFSDLVRYLEYKLLGPETGEGE---GDRG
Sulfolobus           -----EKLKNGNVNYNELNNNFRNLILKKVDEVSKN--------------------EKRK
Archaeoglobus        -----EEWINIAAGKGEA-EYYDFNGIKRVATLD----------------------RQDL
Methanocaldococcus   -----DKIANSEDKKRIYIEYYD-GTRCRXKTVK----------------------KDDE
                        .             :      .   .                         .

Deinococcus          WVLKQSPGTLRAFTRRLRGVQKYLSPLIRGDLTPEQAEGYRPDPLRRGIQLTVVDIHALS
Ocep                 WTARQHTGTLQAFVRRLRAASRSVGHLVRGD-----APSNAPDPLGSPAQVNVVDIHNLS
Tsco                 WVARQARGTLEAFVRRLRSSVENVGHLVRGD-----RKGNPPDPLEGGEQVHVVDLAKLS
JL18                 WTARQAKGTLEAFVRRLKASVENVGHLIRGD-----RPGNPPDPLSGE-QVHVVDLAKLS
HB27                 WTARQAKGTLEAFVRRLRSSVENVAHLVRGD-----RPGSPPDPLSGKAQVHVVDLAKLS
HB8                  WTARQAKGTLEAFVRRLRSSVENVAHLVRGD-----RPGSPPDPLSGKAQVHVVDLAKLS
SG0.5                WTARQAKGTLEAFVRRLRSSVENVAHLVRGD-----RPGSPPDPLSGKAQVHVVDLAKLS
Sulfolobus           DSKDEVINKIEDFLDRY----SEIIDFTAGDVVDKIKIG----------KVNVINLSSLD
Archaeoglobus        NALARIKEYVSSFMRRY----EDL--LSQNDMLANIKPS----------YLNVVNLSGFD
Methanocaldococcus   MSINRVIEKLRWFINKN----KNI--LGENEGMFDIKSD----------KINVLPLQKIE
                              :  *  .       :  :  .:       .           : *::  :.

Deinococcus          AHAQMFVVGVLLREVFEYKE---RVGRQDT---------VFVVLDELNKYAPREGDSPIK
Ocep                 PQAQMFVVGALLRRVFAAKE---SGSHRGK---------VFVVLDELNKYAPREGESPIK
Tsco                 PQGQMFVVGSLLSDLFAKKE---RGQYRGR---------VFVVLDELNKYAPRDEESPIK
```

```
JL18                  PQAQMFVVGSLLKDLFERKE---RGQYRGR---------VFIVLDELNKYAPREEESPIK
HB27                  PQAQMFVVGSLLKDLFERKE---RGQYRGR---------VFIVLDELNKYAPREEESPIK
HB8                   PQAQMFVVGSLLKDLFERKE---RGQYRGR---------VFIVLDELNKYAPREEESPIK
SG0.5                 PQAQMFVVGSLLKDLFERKE---RGQYRGR---------VFIVLDELNKYAPREEESPIK
Sulfolobus            EDAIDAIVSHYLRKILTSRK---ENKMKRKIGLK---FPVLVVIEEAHVLLSKDSNTLTK
Archaeoglobus         EGQMRVVVAYLLRNLLVGRINYVRGKKGWEKICPAIVKPLLVIFEEGHIFAPKGVNNDVV
Methanocaldococcus    DEEAITIVGEFLKRVLKERI---KSVHDEIVEIKALEKPTLVIIEEAHLFAAKNLKDRSG
                         :*.   *   ::   .                 :::::* :    ..   .

Deinococcus           DVLLDIAERGRSLGIILIGAQQTASEVERRIVSNAAIRVVGRLDLAEAER---PEYRFLP
Ocep                  DILLDIAERGRSLGVILIGAQQTASEVERRVVGNAAVRVVGRLDAAEAER---PEYRYLP
Tsco                  DVLLDIAERGRSLGVILIGAQQTASEVERRVVGNAAIRVVGRLDAAEAER---PEYRYLP
JL18                  DVLLDIAERGRSLGVILIGAQQTASEVERRVVGNAAIRVVGRLDAAEAER---PEYRYLP
HB27                  DVLLDIAERGRSLGVILIGAQQTASEVERRVVGNAAIRVVGRLDAAEAER---PEYRYLP
HB8                   DVLLDIAERGRSLGVILIGAQQTASEVERRVVGNAAIRVVGRLDAAEAER---PEYRYLP
SG0.5                 DVLLDIAERGRSLGVILIGAQQTASEVERRVVGNAAIRVVGRLDAAEAER---PEYRYLP
Sulfolobus            HWAGRIAREGRKFGVGLIIVSQRPKGIDENILSQMTNKIILKMVEPSDKKYVLETSDNLS
Archaeoglobus         SWMGRIAREGRKFGIGLGIVSQRPKRLNDDVLSQCNTKIILRITEPNDQRYVQHASEQIS
Methanocaldococcus    YWINRIAKEGRKFGVGLGLVSQRPKELNPTVLSQMNTKIILRIVEPTDQKYILESSENVG
                        **   **.:*: *   ..* .. ::   ::.:    .:: :   .   :.         :

Deinococcus           QSFRGRAGILQPGTMLVSQPDVPNPVLVNYP-------------FPAWATRRDEVDDLGG
Ocep                  GAFRDRALILPQGTMILHQPEVPVPIAVQFP-------------YPAWATKAGEAAE--D
Tsco                  TSFRQRALILPQGMVILHQPEIPVPLLVRFP-------------FPAWATKREEVLE--D
JL18                  TSFRQRALILPQGAVILHQPEIPVPLLVHFP-------------FPAWATKREEVLE--D
HB27                  TSFRQRALILPQGAVILHQPEIPVPLLVHFP-------------FPAWATKREEVLE--D
HB8                   TSFRQRALILPQGAVILHQPEIPVPLLVHFP-------------FPAWATKREEVLE--D
SG0.5                 TSFRQRALILPQGAVILHQPEIPVPLLVHFP-------------FPAWATKREEVLE--D
Sulfolobus            EDIVEGLSALDTGEAVIVGNIVRMPAIVKIDKFEGKLAGSDPNLIEEWKKAKEEIEE--H
Archaeoglobus         EDLLKDIASLGVGEAVVVGPAVKLPVAVKIRKFAGNYGGRDIDVVEEWLGREEEQKK--Q
Methanocaldococcus    EDLLQDLPQLSTGEAIVVGSSLPLPALVKIKKYDGVLGG--------------------E
                      :        *  *   ::     :  *   *.

Deinococcus           KAAAEVGAGLLR
Ocep                  VSDEALKDELGL
Tsco                  NSAEALRREFF-
JL18                  NSAEALRREFFG
HB27                  NSAEALRREFFG
HB8                   NSAEALRREFFR
SG0.5                 NSAEALRREFFG
Sulfolobus            ADVLNWGE----
Archaeoglobus         VTLEDLAV----
Methanocaldococcus    DGLKNLKI----
```

**Figure S1.** Sequence alignments of HepA homologs. CLUSTAL multiple sequence alignment by MUSCLE (3.8). Alignments of the amino acid sequence of HerA homologues in *Deinococcus radiodurans* (Deinocccus), *Oceanithermus profundus* (Ocep), the HepA proteins of *Thermus scotoductus* SA01 (Tsco), *T. thermophilus* strains JL18, HB27, HB8, and SG0.5, and the archaea *Sulfolobus tokodaii* (Sulfolobus), *Archaeoglobus fulgidus* (Archaeoglobus), and *Methanocaldococcus jannaschii* (Methanocaldococcus).
